# Comparison of fluorescence biosensors and whole-cell patch clamp recording in detecting ACh, NE, and 5-HT

**DOI:** 10.3389/fncel.2023.1166480

**Published:** 2023-06-02

**Authors:** Kun Zhang, Yanfei Han, Peng Zhang, Yuqiong Zheng, Aobing Cheng

**Affiliations:** ^1^Department of Pharmacology and Chemical Biology, Shanghai Jiao Tong University School of Medicine, Shanghai, China; ^2^Key Laboratory of Cell Differentiation and Apoptosis of Chinese Ministry of Education, Shanghai Jiao Tong University School of Medicine, Shanghai, China; ^3^Shanghai Frontiers Science Center of Cellular Homeostasis and Human Diseases, Shanghai Jiao Tong University School of Medicine, Shanghai, China; ^4^State Key Laboratory of Oncogenes and Related Genes, Shanghai Cancer Institute, Renji Hospital, Shanghai Jiao Tong University School of Medicine, Shanghai, China; ^5^Department of Anesthesiology, Guangzhou First People’s Hospital, South China University of Technology, Guangzhou, Guangdong, China

**Keywords:** genetically encoded sensors, whole-cell patch clamp recording, acetylcholine (ACh), norepinephrine (NE), serotonin

## Abstract

The communication between neurons and, in some cases, between neurons and non-neuronal cells, through neurotransmission plays a crucial role in various physiological and pathological processes. Despite its importance, the neuromodulatory transmission in most tissues and organs remains poorly understood due to the limitations of current tools for direct measurement of neuromodulatory transmitters. In order to study the functional roles of neuromodulatory transmitters in animal behaviors and brain disorders, new fluorescent sensors based on bacterial periplasmic binding proteins (PBPs) and G-protein coupled receptors have been developed, but their results have not been compared to or multiplexed with traditional methods such as electrophysiological recordings. In this study, a multiplexed method was developed to measure acetylcholine (ACh), norepinephrine (NE), and serotonin (5-HT) in cultured rat hippocampal slices using simultaneous whole-cell patch clamp recordings and genetically encoded fluorescence sensor imaging. The strengths and weaknesses of each technique were compared, and the results showed that both techniques did not interfere with each other. In general, genetically encoded sensors GRAB_*NE*_ and GRAB_5*HT*1_._0_ showed better stability compared to electrophysiological recordings in detecting NE and 5-HT, while electrophysiological recordings had faster temporal kinetics in reporting ACh. Moreover, genetically encoded sensors mainly report the presynaptic neurotransmitter release while electrophysiological recordings provide more information of the activation of downstream receptors. In sum, this study demonstrates the use of combined techniques to measure neurotransmitter dynamics and highlights the potential for future multianalyte monitoring.

## Introduction

Neurons and glial cells are the key components of the human brain. As the basic working units of the brain, neurons communicate with one another through synapses, with most inter-neuronal communication taking place at chemical synapses (Majhi et al., 2019). An action potential arriving at the axon terminal causes the cell membrane to depolarize and opens voltage-gated calcium channels, initiating a signaling cascade that leads to the release of neurotransmitters into the synaptic cleft (Luo, 2020). These released neurotransmitters then diffuse across the synaptic cleft and bind to receptors on the postsynaptic membrane, causing the postsynaptic membrane to either depolarize or hyperpolarize, which allows for the transfer of information between neurons or the alteration of the target neuron’s cellular properties (Sudhof, 2008, 2013; Kaeser and Regehr, 2014; Luo, 2020). The proper regulation of neurotransmission is critical for the normal functioning of the central nervous system. Conversely, dysregulation of neurotransmission has been implicated in a variety of neurological and psychiatric conditions, such as Alzheimer’s disease (AD), Parkinson’s disease (PD), depression, autism, schizophrenia, and addiction (Francis et al., 1999; Sarter et al., 2007; Ballatori et al., 2009). However, the synaptic properties of neurotransmission are not yet fully understood primarily due to the limitations of available tools for quantitative analysis of different neurotransmitters with high spatial and temporal resolution.

Many experimental methods, including molecular biology, electrophysiology, and imaging, are used to investigate changes in neurotransmission. The patch clamp technique is an essential electrophysiological tool that provides valuable insight into the functional activity of neurons and neurotransmitter dynamics by measuring electrical potentials and currents across the cell membrane, allowing scientists to decode intercellular and intracellular information (Reyes, 2019). Whole-cell electrophysiology has served as the gold standard to delineate synaptic properties of glutamatergic and GABAergic transmission because of its ability to functionally dissect synaptic transmission at molecular, cellular and network levels with remarkable sensitivity and high spatiotemporal localization (Neher, 2015; Jackman and Regehr, 2017). However, electrophysiology recordings acquire information from discrete electrode and only a limited number of electrodes could be applied simultaneously due to constrained space of the electrodes. For instance, although octuple patch-clamp recording has been developed to dissect neural circuits, they are technically challenging and are largely restricted to *in vitro* brain slice studies (Jiang et al., 2013; Wang et al., 2015), which hinders its wide use. Even with multiple-electrode arrays, researchers could only record at most hundreds of neurons at a time. Additionally, the technique may not be suitable for cells with minimal or no neurotransmitter-induced responses (Dani and Bertrand, 2007; Nadim and Bucher, 2014). However, a deep understand of neurotransmission in brains requires the researchers to capture as much information (ideally all brain cells) as possible simultaneously. To overcome these limitations, researchers have developed new tools to monitor neurotransmitters with high sensitivity, specificity, and high spatial and temporal resolution. Over the last decade, two major groups of genetically encoded neurotransmitter sensors have been developed, including G-protein-coupled receptor (GPCR) and bacterial periplasmic binding protein (PBP) sensors for glutamate (Marvin et al., 2013), GABA (Marvin et al., 2019), dopamine (Patriarchi et al., 2018; Sun et al., 2018, 2020), acetylcholine (Jing et al., 2018; Borden et al., 2020), norepinephrine (Feng et al., 2019), and serotonin (Unger et al., 2020; Wan et al., 2021). These sensors have been shown to effectively and specifically detect endogenous neurotransmitters in neuronal and non-neuronal tissue preparations *in vitro*, *ex vivo*, and *in vivo* (Zhu et al., 2020; Chen et al., 2021; Dong et al., 2022; Swanson et al., 2022).

The aim of this study was to compare the advantages and disadvantages of classical electrophysiology methods and newly developed genetically encoded neurotransmitter sensors. We chose acetylcholine (ACh), norepinephrine (NE), and serotonin (5-HT) in current study because they all belong to neuromodulatory transmitter systems and share a number of common features (Gu, 2002). Beyond the central nervous system, neuromodulatory transmitters are found and participate in many other tissue and organ functions (Berger et al., 2009; Dantzer, 2018). And deregulation of peripheral and non-neuronal cholinergic/adrenergic/serotoninergic signals is implicated in many non-neuronal pathological conditions, including cancer, diabetes, and cardiovascular diseases (Berger et al., 2009; Kruse et al., 2014; Herring et al., 2019; Sarrouilhe and Mesnil, 2019; Ye et al., 2021). We showed that both whole-cell patch clamp recordings and genetically encoded sensors (iAChSnFR, GRAB_*NE*_, and GRAB_5*HT*1_._0_) can be used in tandem in rat hippocampal slices without any interference. This comparison highlights the strengths and limitations of both approaches, providing researchers with guidance on the most appropriate tools for their specific needs and scientific goals.

## Materials and methods

### Animals

Wild type male/female SD rats were used in this study. All rats were housed in a temperature-controlled room with a 12/12 h light/dark cycle, with humidity controlled as 55%, provided with food and water *ad libitum*. All procedures for animal surgery and maintenance were performed using protocols that were approved by the Animal Care and Use Committees at Shanghai Jiao Tong University School of Medicine (SYXK(Hu)2018-0027).

### Cultured slice preparation

Organotypic hippocampal cultured slices were prepared from postnatal 5–7 days old male/female SD rats following our previous studies (Jing et al., 2018; Borden et al., 2020). Briefly, the hippocampi were dissected out in ice-cold HEPES-buffered Hanks’ solution (pH 7.35) under sterile conditions, sectioned into 400 μm slices on a tissue chopper, and explanted onto a Millicell-CM membrane (0.4 μm pore size; Millipore, Billerica, MA, USA). The membranes were then placed in 750 μl of MEM culture medium, containing (in mM): HEPES 30, heat-inactivated horse serum 20%, glutamine 1.4, D-glucose 16.25, NaHCO_3_ 5, CaCl_2_ 1, MgSO_4_ 2, insulin 1 mg/ml, ascorbic acid 0.012% at pH 7.28 and osmolarity 320. Cultured slices were maintained at 35°C in a humidified incubator (ambient air enriched with 5% CO_2_).

### Sindbis virus preparation and expression of genetically encoded sensors

Genetically encoded ACh, NE, and 5-HT fluorescent sensors were gifts from Dr. Julius Zhu at University of Virginia and then sub-cloned into Sindbis construct, and viral particles were produced following previous studies (Jing et al., 2018). In brief, fluorescent sensors were sub-cloned into Sindbis viral vector pSinREP5 with Xbal and Sphl restriction digestion. Expression of genetically encoded fluorescent sensors was performed as previously reported (Jing et al., 2018; Zhang et al., 2018, 2022). A glass pipette was used to penetrate the cultured hippocampal slices to deliver ∼50 nl of Sindbis solution by pressure injection to CA1, CA3, or DG cell layer to infect only a few CA1, CA3, or DG neurons (i.e., 1–5 neurons per slice/brain) as indicated. Experiments were performed 18 ± 4 h after Sindbis viral infection.

### Simultaneous sensor imaging and whole-cell patch clamp recording

For the Sindbis virus-infected cultured rat hippocampal slices, wide-field epifluorescence imaging was performed using Hamamatsu ORCA FLASH4.0 camera (Hamamatsu Photonics, Japan), and sensor-expressing cells in cultured brain slices are excited by a 460-nm ultra-high power low-noise LED (Prizmatix, Givat-Shmuel, Israel). The patch recording pipettes (4-7 MΩ) were filled with intracellular solution 120 mM potassium gluconate, 4 mM KCl, 10 mM HEPES, 4 mM MgATP, 0.3 mM Na_3_GTP, and 10 mM sodium phosphocreatine (pH 7.25) for voltage-clamp recordings. Bath solution (29 ± 1.5°C) contained (in mM): NaCl 119, KCl 2.5, CaCl_2_ 4, MgCl_2_ 4, NaHCO_3_ 26, NaH_2_PO_4_ 1, and glucose 11, at pH 7.4 and gassed with 5% CO_2_/95% O_2_. Dual whole-cell recordings were obtained from two nearby infected and non-infected hippocampal CA1/CA3 pyramidal neurons with two Axopatch-200B patch clamp amplifiers (Molecular Devices, Sunnyvale, CA, USA) under visual guidance using fluorescence and transmitted light illumination. Sensor-expressing neurons were patch-clamp recorded and loaded with 5 μM Alexa Fluor 594 (Life Technologies) for ∼10 min to verify that imaged cells and patched cells were the same. Agonists, including ACh, NE, and 5-HT (Tocris Bioscience, Bristol, UK), were puff-applied with a glass pipette (1–1.5 μm in tip diameter) positioned ∼50 μm above the imaged neurons using 10 ms 30-kPa pressure pulses. The frame rate of the FLASH4.0 camera was set to 10 Hz and the sampling rate of electrophysiological recording was set to 10 KHz. To synchronize sensor image capture and whole-cell patch clamp recordings with puff experiments, the camera was set to external trigger mode and triggered by a custom-written IGOR Pro 6 program (WaveMetrics, Lake Oswego, OR, USA).

### Statistical analysis

All results are reported as mean ± SEM. Animals or cells were randomly assigned into control or experimental groups and investigators were blinded to experiment treatments in cultured slices. Statistical significance of the means (*p* < 0.05; two sides) was determined using Student’s *t*-test or one-way ANOVA with Dunnett’s *post-hoc* test analysis.

## Results

### Establishment of simultaneous sensor imaging and dual whole-cell recording system

**FIGURE 1:**
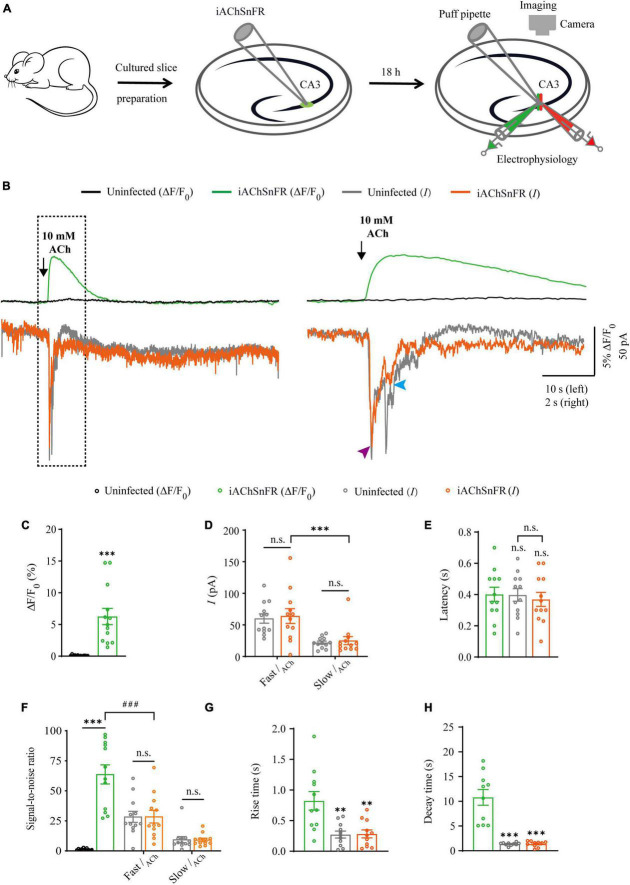
Simultaneous measurement of acetylcholine using iAChSnFR fluorescent sensor and electrophysiological recording in CA3 neurons. **(A)** Schematic drawing of the design of simultaneous sensor imaging and whole-cell patch clamp recording experiments in cultured rat hippocampal slice preparation. **(B)** Left, simultaneous fluorescence (upper panel) and currents (lower panel) of iAChSnFR-expressing and control non-expressing CA3 neurons to a brief puff application of ACh. Right, the responses in the left rectangle box are shown again on an expanded time scale. **(C)** Values for fluorescence responses of iAChSnFR-expressing CA3 neurons compared to non-expressing CA3 neurons (iAChSnFR: 6.25 ± 1.28%; Ctrl: 0.11 ± 0.02%; *p* < 0.0010; *n* = 13). **(D)** Values for the amplitudes of fast cholinergic currents (iAChSnFR: 63.92 ± 11.71 pA; Ctrl: 60.06 ± 7.46 pA; *p* = 0.79; *n* = 12) and slow cholinergic currents (iAChSnFR: 25.08 ± 6.36 pA; Ctrl: 21.73 ± 2.51 pA; *p* = 0.67; *n* = 12; Fast current vs. Slow current: *p* = 0.0010) in iAChSnFR-expressing CA3 neurons compared to non-expressing CA3 neurons. **(E)** Values for the latencies of fast cholinergic currents in non-expressing (Ctrl: 0.40 ± 0.04 s; *p* = 1.00; *n* = 12) and iAChSnFR-expressing (iAChSnFR: 0.37 ± 0.04 s; *p* = 0.82; *n* = 12; Ctrl current vs. iAChSnFR current: *p* = 0.85) CA3 neurons compared to those of fluorescence responses of iAChSnFR-expressing CA3 neurons (iAChSnFR: 0.40 ± 0.05 s; *n* = 12). **(F)** Values for the signal-to-noise ratio (SNR) of cholinergic fluorescence responses of iAChSnFR-expressing CA3 neurons compared to non-expressing CA3 neurons (iAChSnFR: 63.67 ± 7.94; Ctrl: 1.34 ± 0.23; *p* < 0.0010; *n* = 12), and that of fast (iAChSnFR: 28.47 ± 5.29; Ctrl: 28.36 ± 4.58; *p* = 0.99; *n* = 12) and slow (iAChSnFR: 9.13 ± 1.41; Ctrl: 9.37 ± 2.54; *p* = 0.94; *n* = 12) cholinergic currents of iAChSnFR-expressing CA3 neurons compared to non-expressing CA3 neurons. Values for the signal-to-noise ratio (SNR) of fast cholinergic currents in iAChSnFR-expressing CA3 neurons (iAChSnFR: 28.47 ± 5.29; *p* < 0.0010; *n* = 12) compared to those of fluorescence responses of iAChSnFR-expressing CA3 neurons (iAChSnFR: 63.67 ± 7.94; *n* = 12). **(G)** Values for rise time of the cholinergic currents in non-expressing (Ctrl: 0.27 ± 0.06 s; *p* = 0.0052; *n* = 11) and iAChSnFR-expressing (iAChSnFR: 0.28 ± 0.07 s; *p* = 0.0036; *n* = 11) CA3 neurons compared to those of fluorescence responses of iAChSnFR-expressing CA3 neurons (iAChSnFR: 0.82 ± 0.15 s; *n* = 11). **(H)** Values for decay time of the cholinergic currents in non-expressing (Ctrl: 1.36 ± 0.10 s; *p* < 0.0010; *n* = 10) and iAChSnFR-expressing (iAChSnFR: 1.37 ± 0.19 s; *p* < 0.0010; *n* = 10) CA3 neurons compared to those of fluorescence responses of iAChSnFR-expressing CA3 neurons (iAChSnFR: 10.79 ± 1.59 s; *n* = 10). Data are shown as mean ± SEM. ^***^p < 0.001, ^**^p < 0.01, two-tailed Student’s unpaired *t*-test in panels **(C,D,G,H)**, ^###^*p* < 0.001, one-way ANOVA with Dunnett’s *post-hoc* test in panels **(E,F)**.

**FIGURE 2:**
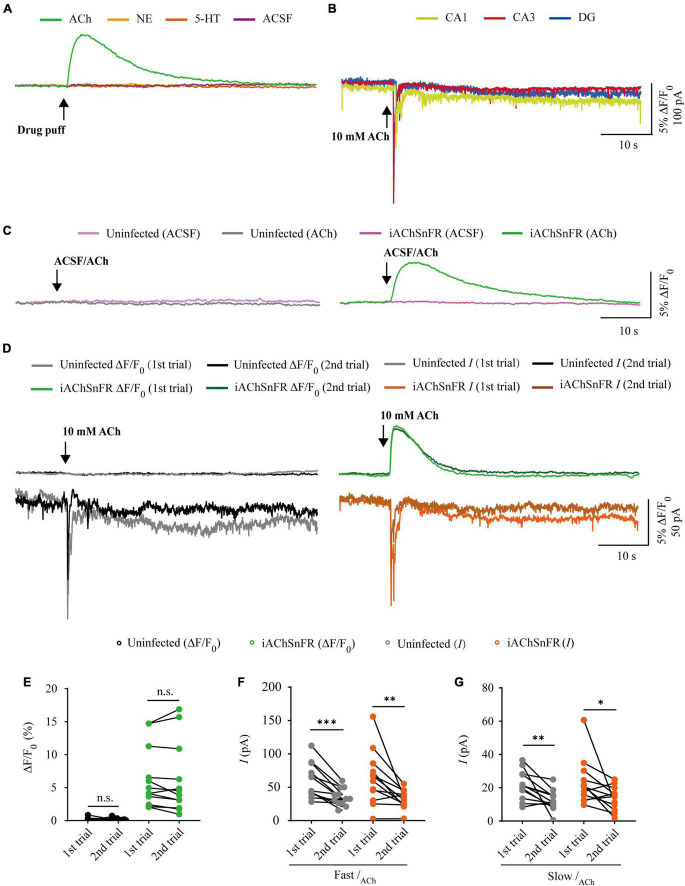
Comparison of iAChSnFR fluorescence and electrophysiological responses to two consecutive acetylcholine application. **(A)** Fluorescence responses of iAChSnFR-expressing cells to a brief drug puff (10 ms) application of 10 mM ACh, 200 μM norepinephrine (NE) and 100 μM serotonin (5-HT), respectively. **(B)** Electrophysiological responses of CA1, CA3, and DG neurons to a brief (10 ms) puff application of 10 mM ACh. **(C)** Fluorescence responses of control non-expressing (left) and iAChSnFR-expressing (right) CA3 neurons to a brief puff (10 ms) application of ACSF and 10 mM acetylcholine (ACh). **(D)** Fluorescence (upper panel) and electrophysiological responses (lower panel) of non-expressing (left) and iAChSnFR-expressing (right) CA3 neurons to two consecutive puff applications of ACh. **(E)** Values for the two consecutive fluorescence responses of non-expressing (first: 0.18 ± 0.07%; second: 0.19 ± 0.06%; *p* = 0.71; *n* = 12) and iAChSnFR-expressing (first: 6.39 ± 1.34%; second: 6.15 ± 1.56%; *p* = 0.38; *n* = 12) CA3 neurons. **(F)** Values for the two consecutive fast cholinergic currents in non-expressing (first: 60.06 ± 7.46 pA; second: 35.26 ± 3.74 pA; *p* < 0.0010; *n* = 12) and iAChSnFR-expressing (first: 63.92 ± 11.71 pA; second: 32.29 ± 4.02 pA; *p* = 0.0094; *n* = 12) CA3 neurons. **(G)** Values for the two consecutive slow cholinergic currents of non-expressing (first: 21.73 ± 2.51 pA; second: 12.48 ± 1.76 pA; *p* = 0.0063; *n* = 12) and iAChSnFR-expressing (first: 23.42 ± 4.00 pA; second: 13.67 ± 2.06 pA; *p* = 0.012; *n* = 12) CA3 neurons. Data are shown as mean ± SEM. ^***^p < 0.001, ^**^p < 0.01, *p < 0.05, two-tailed Student’s paired *t*-test.

**FIGURE 3:**
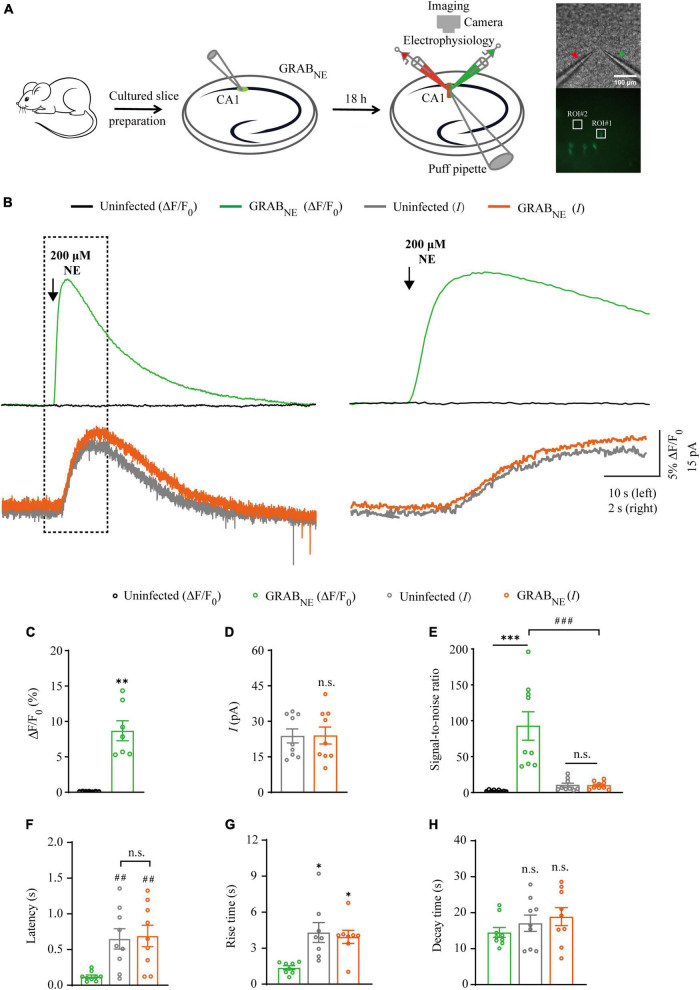
Simultaneous measurement of norepinephrine using GRAB_NE_ fluorescent sensor and electrophysiological recording in CA1 neurons. **(A)** Schematic drawing of the design of simultaneous imaging and electrophysiological recording experiments in cultured rat hippocampal slices. The red arrow indicates the patch electrode of control cell and green arrow indicates that of sensor-expressing cell. The white squares indicating regions of interest (ROIs). **(B)** Left, simultaneous fluorescence (upper panel) and electrophysiological (lower panel) responses of control non-expressing and GRAB_*NE*_-expressing CA1 neurons to a brief puff application of NE. Right, the responses in the left rectangle box are shown again on an expanded time scale. **(C)** Values for the fluorescence responses of GRAB_*NE*_-expressing CA1 neurons compared to non-expressing CA1 neurons (GRAB_NE_: 8.68 ± 1.41%; Ctrl: 0.12 ± 0.01%; *p* < 0.0010; *n* = 7). **(D)** Values for the amplitudes of adrenergic currents in GRAB_NE_ expressing CA1 neurons compared to non-expressing CA1 neurons (GRAB_NE_: 23.80 ± 2.95 pA; Ctrl: 23.99 ± 3.59 pA; *p* > 0.99; *n* = 9). **(E)** Values for the signal-to-noise ratio (SNR) of fluorescence responses of GRAB_*NE*_-expressing CA1 neurons compared to non-expressing CA1 neurons (GRAB_NE_: 92.61 ± 19.87; Ctrl: 2.43 ± 0.39; *p* < 0.0010; *n* = 9), and that of adrenergic currents of GRAB_*NE*_-expressing CA1 neurons compared to non-expressing CA1 neurons (GRAB_NE_: 9.78 ± 1.80; Ctrl: 10.15 ± 2.80; *p* = 0.67; *n* = 9). Values for the SNR of adrenergic currents in GRAB_*NE*_-expressing CA1 neurons (9.78 ± 1.80; *p* < 0.0010; *n* = 9) compared to those of the fluorescence responses of GRAB_*NE*_-expressing CA1 neurons (92.61 ± 19.87; *n* = 9). **(F)** Values for the latencies of adrenergic currents in non-expressing (Ctrl: 0.65 ± 0.14 s; *p* = 0.0091; *n* = 9) and GRAB_*NE*_-expressing (GRAB_NE_: 0.69 ± 0.15 s; *p* = 0.0051; *n* = 9; Ctrl vs. GRAB_NE_ current: *p* = 0.96; *n* = 9) CA1 neurons compared to those of fluorescence responses of GRAB_*NE*_-expressing CA1 neurons (GRAB_NE_: 0.12 ± 0.02 s; *n* = 9). **(G)** Values for rise time of the adrenergic currents in non-expressing (Ctrl: 4.31 ± 0.82 s; *p* = 0.014; *n* = 8) and GRAB_*NE*_-expressing (GRAB_NE_: 3.94 ± 0.55 s; *p* = 0.016; *n* = 8) CA1 neurons compared to those of fluorescence responses of GRABNE-expressing CA1 neurons (GRAB_NE_: 1.37 ± 0.17 s; *n* = 8). **(H)** Values for decay time of the adrenergic currents in non-expressing (Ctrl: 17.08 ± 2.27 s; *p* = 0.21; *n* = 9) and GRAB_*NE*_-expressing (GRAB_NE_: 18.90 ± 2.50 s; *p* = 0.086; *n* = 9) neurons compared to those of fluorescence responses of GRAB_*NE*_-expressing CA1 neurons (GRAB_NE_: 14.50 ± 1.38 s; *n* = 9). Data are shown as mean ± SEM. ^***^p < 0.001, ^**^p < 0.01, *p < 0.05, two-tailed Student’s unpaired *t*-test in panels **(C,D,G,H)**, ^###^*p* < 0.001, ^##^*p* < 0.01, one-way ANOVA with Dunnett’s *post-hoc* test in panels **(E,F)**.

**FIGURE 4:**
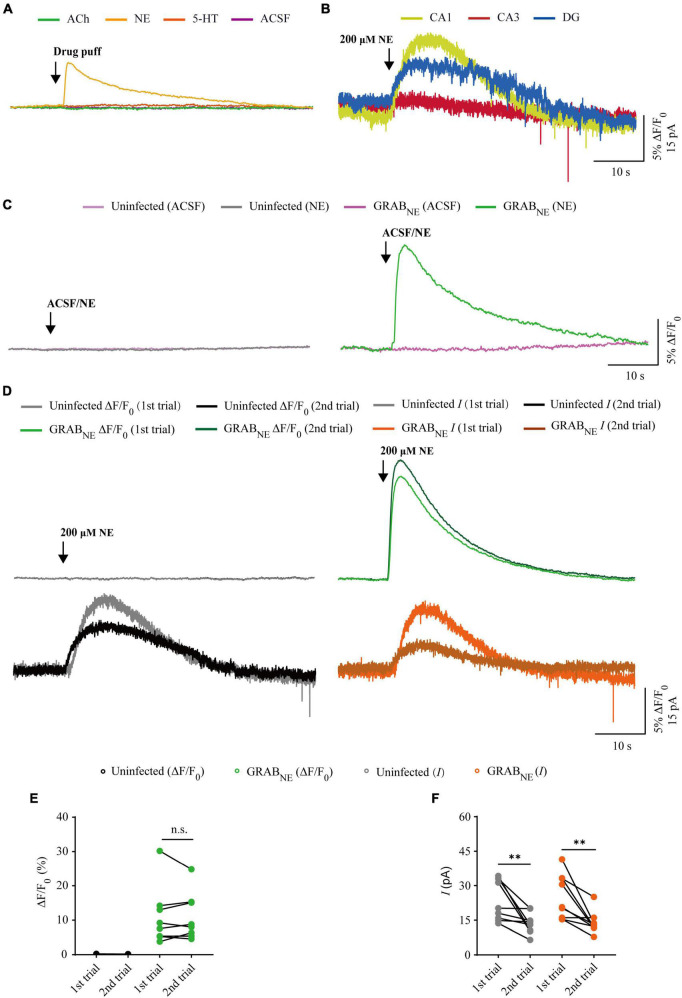
Comparison of GRAB_NE_ fluorescence and electrophysiological responses to two consecutive norepinephrine application. **(A)** Fluorescence responses of GRAB_*NE*_-expressing cells to a brief drug puff (10 ms) application of 200 μM NE, 10 mM ACh, 100 μM 5-HT and ACSF, respectively. **(B)** Electrophysiological responses of CA1, CA3, and DG neurons to a brief (10 ms) puff application of 200 μM NE. **(C)** Fluorescence responses of control non-expressing (left) and GRAB_*NE*_-expressing (right) CA1 neurons to a brief puff (10 ms) application of ACSF and 200 μM NE. **(D)** Fluorescence (upper panel) and electrophysiological (lower panel) responses of non-expressing (left) and GRAB_*NE*_-expressing (right) CA1 neurons to two consecutive puff (10 ms) of 200 μM NE. **(E)** Values for the two consecutive fluorescence responses of non-expressing (first: 0.13 ± 0.02%; second: 0.11 ± 0.01%; *p* = 0.10; *n* = 9) and GRAB_*NE*_-expressing (first: 10.45 ± 2.75%; second: 10.41 ± 2.43%; *p* = 0.65; *n* = 9) CA1 neurons. **(F)** Values for the two consecutive adrenergic currents in non-expressing (first: 23.80 ± 2.95 pA; second: 13.63 ± 1.48 pA; *p* = 0.0039; *n* = 9) and GRAB_*NE*_-expressing (first: 25.10 ± 3.21 pA; second: 13.75 ± 1.59 pA; *p* = 0.0078; *n* = 9) CA1 neurons. Data are shown as mean ± SEM. ^**^p < 0.01, two-tailed Student’s paired *t*-test.

Whole-cell patch clamp has proven ideal for studying the collective properties of neurons and neuronal networks in brain slices maintained *in vitro*, whereas recently developed genetically encoded sensors enabled us to directly visualize neurotransmitter/neuromodulator release with high temporal and spatial resolution. To directly compare the performance of the whole-cell patch clamp method and genetically encoded sensors in reporting neuromodulatory neurotransmitter release, we established a simultaneous electrophysiological recording and fluorescence imaging system. First, we expressed genetically encoded sensors in CA1/CA3 pyramidal neurons in cultured rat hippocampal slices with the Sindbis viral expression system that permitted a more rapid (∼18 h) and robust expression of the sensors (Jing et al., 2018). Next, we directly delivered specific neurotransmitters at the indicated concentration with an air puff system to evoke fluorescence and electrophysiological responses. The fluorescence responses captured by an epifluorescence microscope and whole-cell patch clamp recording showed that a brief 10 ms puff application of neurotransmitters at optimized concentrations could evoke both fluorescence response and membrane currents in the sensor expressing neurons (Figures 1B, 2B, 3B), whereas puff application of the control bath solution artificial cerebrospinal fluid (ACSF) induced no responses in the same neurons (Figures 2C, 4C). From here on, we applied this system to directly compare the properties of sensors and electrophysiological recordings in detecting ACh, NE, and 5-HT.

### Comparison of iAChSnFR sensor and electrophysiology in probing ACh signals

There are two types of genetically encoded fluorescent sensors for ACh, including GACh2.0/GACh3.0 (Jing et al., 2018, 2020) and iAChSnFR (Borden et al., 2020). GACh2.0 and GACh3.0 are constructed based on human muscarinic acetylcholine receptor 3 (hM3R), with circularly permutated GFP (cpGFP) inserted into the third intracellular loop (ICL3) of hM3R; whereas iAChSnFR is constructed using bacterial periplasmic binding protein (PBP) as the ACh recognition moiety and cpGFP as the fluorescent reporter module (Jing et al., 2018; Borden et al., 2020). Previously, it was reported that iAChSnFR showed higher sensitivity and faster on/off kinetics (Borden et al., 2020). Therefore, we chose iAChSnFR as the genetically encoded ACh sensor for the subsequent experiments. To directly compare iAChSnFR sensor with traditional electrophysiological recordings, we simultaneously made whole-cell recording and fluorescence imaging from pairs of neighboring iAChSnFR-expressing and control non-infected CA3 pyramidal neurons (Figure 1A), which had a robust current response to cholinergic stimulation in cultured hippocampal slices (Figure 2B), which is consistent with what has reported before (Grybko et al., 2010). A 10 ms ACh puff evoked a brief, large inward current (Figure 1B, pink arrow) followed by a prolonged, small inward current (Figure 1B, blue arrow) in both iAChSnFR-expressing and non-expressing neurons, presumably representing the activation of endogenous nicotinic and muscarinic receptors, respectively (Figures 1B, D). A concurrent fluorescence signal was observed only in iAChSnFR-expressing neurons but not in the control non-expressing CA3 neurons (Figures 1B, C, 2C). The latencies of the fast cholinergic currents and the fluorescence responses were the same in iAChSnFR-expressing neurons (Figures 1B, E), indicating that iAChSnFR could detect ACh as fast as endogenous cholinergic receptors. Furthermore, the signal-to-noise ratio (SNR) was calculated as the peak response dividing the standard error of baseline fluorescence/current. The SNR of the iAChSnFR fluorescence responses (∼60) seemed to be larger than that of the fast nicotinic-like cholinergic currents (∼28), and slow muscarinic-like cholinergic currents (∼9) (Figures 1B, F). To further compare the temporal responses, we compared rise time (10–90%) to the maximal responses and decay time (time from maximum to 50%) and found that iAChSnFR showed both much slower rise and decay time when compared to cholinergic currents (Figures 1G, H). This slow kinetic feature of iAChSnFR sensor may limit its use for accurately capturing high-frequency induced ACh release. Moreover, there was no difference in the amplitude, latency, SNR, rise, or decay time of the cholinergic currents in iAChSnFR-expressing and control non-expressing neurons (Figures 1D–H), further confirming that the expression of iAChSnFR did not affect the intrinsic properties of CA3 neurons. We further checked the specificity of iAChSnFR and found that iAChSnFR only showed fluorescent response to puffed ACh, but not NE, 5-HT, or control ACSF (Figure 2A). Moreover, we only detected fluorescent response in iAChSnFR-expressing neurons, but not nearby control uninfected neurons to puffed ACh (Figure 2C), confirming the good specificity of iAChSnFR. To further check the stability of iAChSnFR sensor and whole-cell recording in detecting ACh, we compared iAChSnFR fluorescence and electrophysiological responses to two consecutive ACh applications with an 8-min time interval. The second ACh puff evoked the same fluorescence responses, but smaller cholinergic currents (reduced by ∼40%) in iAChSnFR-expressing neurons compared to the first puff (Figures 2D–G), due presumably to the desensitization and endocytosis of endogenous receptors (Dani and Bertrand, 2007). These results indicated that iAChSnFR had better performance in monitoring presynaptic ACh signals repeatedly over long periods, while whole cell recordings could show the cell state of downstream receptor desensitization.

### Comparison of GRAB_*NE*_ sensor and electrophysiology in probing NE signals

There is only one type of genetic fluorescent sensor for NE, including GRAB_*NE*1*m*_ [with a half maximal effective concentration (EC_50_) of 930 nM and a maximum ΔF/F_0_ of approximately 230% in response to a saturating concentration of NE] and GRAB_*NE*1*h*_ (with EC_50_ of ∼83 nM and a maximum ΔF/F_0_ of approximately 130%) (Feng et al., 2019), both of which are constructed based on the human α2 adrenergic G-protein-coupled receptor (α2AR). We chose GRAB_*NE*1*m*_ (from here on, GRAB_*NE*_ in brief) as the genetically encoded NE sensor for the subsequent experiments since it exhibited better performance with respect to ΔF/F_0_ and brightness (Feng et al., 2019). To evaluate the performance of GRAB_*NE*_ sensor and electrophysiological recording in detecting NE signal, we made simultaneous dual whole-cell recordings and fluorescence imaging in pairs of neighboring GRAB_*NE*_-expressing and non-expressing CA1 pyramidal neurons (Figure 3A), which had larger current response (Figure 4B) to adrenergic stimulation compared to other regions in cultured hippocampal slices (Murchison et al., 2011). A 10 ms puff of 200 μM NE evoked a large outward current in both GRAB_*NE*_-expressing and non-expressing neurons, presumably representing the activation of G-protein-coupled inwardly rectifying potassium channels (Figures 3B, D). A concurrent fluorescence signal was observed only in GRAB_*NE*_-expressing neurons, but not in control non-expressing CA1 neurons (Figures 3B, C). The SNR of the GRAB_*NE*_ fluorescence responses (∼90) was larger than that of the adrenergic currents (∼10) (Figure 3E). The latencies of the adrenergic currents were longer than those of the fluorescence responses in GRAB_*NE*_-expressing neurons (Figure 3F), indicating that GRAB_*NE*_ could detect NE signals faster than endogenous adrenergic receptors. To further compare the temporal responses, we compared rise time and decay time and found that GRAB_*NE*_ showed both much faster rise but comparable decay time to adrenergic currents of metabotropic receptors (Figures 3G, H), suggesting a relatively faster response time and better kinetics of GRAB_*NE*_ over whole-cell patch clamp in reporting NE signals. Moreover, there was no difference in the amplitude, latency, SNR, rise time or decay time of adrenergic currents in GRAB_*NE*_-expressing and the neighboring non-expressing control neurons (Figures 3D–H), indicating that the expression of GRAB_*NE*_ had no obvious effect on the physiological properties of CA1 neurons. We further checked the specificity of GRAB_*NE*_ and found that this sensor only showed fluorescent response to puffed NE, but not ACh, 5-HT, or control ACSF (Figure 4A). Moreover, we only detected fluorescent response in GRAB_*NE*_-expressing neurons to puffed NE, but not nearby control uninfected neurons to NE/ACSF (Figure 4C), confirming the good specificity of GRAB_*NE*_. To further check the stability of both methods, we compared fluorescence and electrophysiological responses to two consecutive NE applications. Similarly, the second NE puff evoked almost the same fluorescence response, but smaller adrenergic currents (reduced by ∼45%) in GRAB_*NE*_-expressing neurons compared to the first puff (Figures 4D–F), due presumably to the desensitization of endogenous receptors (Benarroch, 2009), suggesting the suitability of GRAB_*NE*_ in monitoring NE signals repeatedly over long periods.

### Comparison of GRAB_5*HT*1_._0_ sensor and electrophysiology in probing 5-HT signals

**FIGURE 5:**
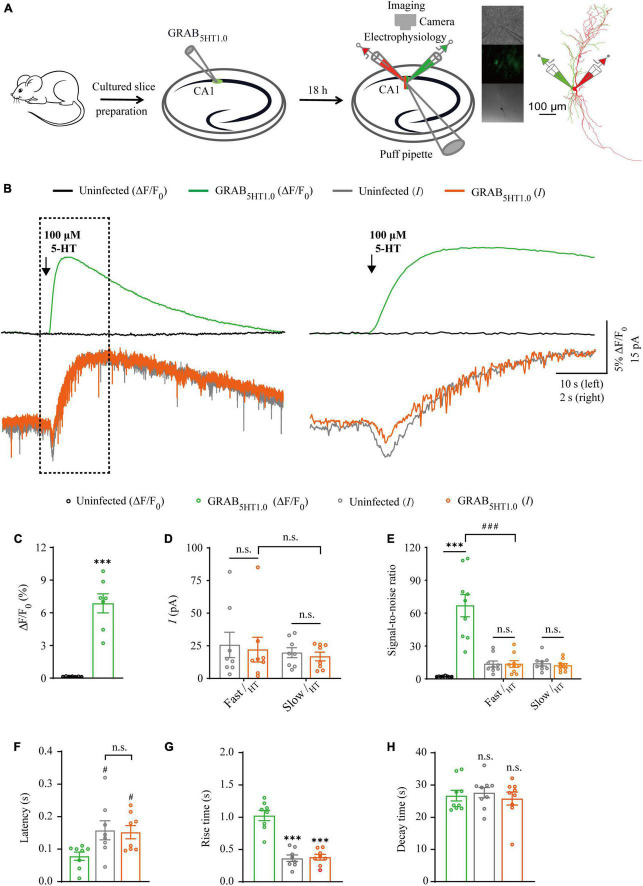
Simultaneous measurement of serotonin using GRAB_5HT1_._0_ fluorescent sensor and electrophysiological recording in CA1 neurons. **(A)** Schematic drawing outlining the design of simultaneous sensor imaging and electrophysiological recording experiments in cultured rat hippocampal slices. **(B)** Left, simultaneous fluorescence (upper panel) and electrophysiological (lower panel) responses of neighboring paired non-expressing and GRAB_5HT1_._0_-expressing CA1 neurons to a brief puff application of 5-HT. Right, the responses in the left rectangle box are shown again in an expanded time scale. **(C)** Values for the fluorescence responses of GRAB_5HT1_._0_-expressing CA1 neurons compared to non-expressing CA1 neurons (GRAB_5HT1_._0_: 6.86 ± 0.88%; Ctrl: 0.15 ± 0.01%; *p* < 0.0010; *n* = 7). **(D)** Values for the amplitudes of fast (GRAB_5HT1_._0_: 22.02 ± 9.45 pA; Ctrl: 25.66 ± 9.75 pA; *p* = 0.96; *n* = 8) and slow serotoninergic currents (GRAB_5HT1_._0_: 16.80 ± 3.32 pA; Ctrl: 19.69 ± 3.83 pA; *p* = 0.57; *n* = 8; Fast current vs. Slow current: *p* > 0.99) in GRAB_5HT1_._0_-expressing CA1 neurons compared to non-expressing CA1 neurons. **(E)** Values for the signal-to-noise ratio (SNR) of serotoninergic fluorescence responses of GRAB_5HT1_._0_-expressing CA1 neurons compared to non-expressing CA1 neurons (GRAB_5HT1_._0_: 66.85 ± 10.14; Ctrl: 2.16 ± 0.22; *p* < 0.0010; *n* = 9), and that of fast (GRAB_5HT1_._0_: 13.58 ± 3.04; Ctrl: 13.60 ± 2.72; *p* = 1.00; *n* = 9) and slow (GRAB_5HT1_._0_: 12.17 ± 1.81; Ctrl: 13.94 ± 2.23; *p* = 0.56; *n* = 9) serotoninergic currents of GRAB_5HT1_._0_-expressing CA1 neurons compared to non-expressing CA1 neurons. Values for the SNR of fast serotoninergic currents in GRAB_5HT1_._0_-expressing CA1 neurons (current: 13.58 ± 3.04; *p* < 0.0010; *n* = 9) compared to those of fluorescence responses of GRAB_5HT1_._0_-expressing CA1 neurons (fluorescence: 66.85 ± 10.14; *n* = 9). **(F)** Values for latencies of fast serotoninergic currents in non-expressing (Ctrl: 0.16 ± 0.03 s; *p* = 0.032; *n* = 8) and GRAB_5HT1_._0_-expressing (GRAB_5HT1_._0_: 0.15 ± 0.02 s; *p* = 0.047; *n* = 8; Ctrl current vs. GRAB_5HT1_._0_ current: *p* = 0.98) CA1 neurons compared to those of fluorescence responses of GRAB_5HT1_._0_-expressing CA1 neurons (GRAB_5HT1_._0_: 0.08 ± 0.01 s, *n* = 8). **(G)** Values for rise time of the serotoninergic currents in non-expressing (Ctrl: 0.36 ± 0.05 s; *p* < 0.0010; *n* = 8) and GRAB_5HT1_._0_-expressing (GRAB_5HT1_._0_: 0.38 ± 0.04 s; *p* < 0.0010; *n* = 8) CA1 neurons compared to those of fluorescence responses of iAChSnFR-expressing CA1 neurons (GRAB_5HT1_._0_: 1.00 ± 0.08 s; *n* = 8). **(H)** Values for decay time of the serotoninergic currents in non-expressing (Ctrl: 27.65 ± 1.60 s; *p* = 0.57; *n* = 9) and GRAB_*NE*_-expressing (GRAB_5HT1_._0_: 25.82 ± 2.02 s; *p* = 0.91; *n* = 9) CA1 neurons compared to those of fluorescence responses of GRAB_*NE*_-expressing CA1 neurons (GRAB_5HT1_._0_: 26.73 ± 1.65 s; *n* = 9). Data are shown as mean ± SEM. ^***^p < 0.001, two-tailed Student’s unpaired *t*-test in panels **(C,D,G,H)**, ^###^*p* < 0.001, ^#^*p* < 0.05, one-way ANOVA with Dunnett’s *post-hoc* test in panels **(E,F)**.

**FIGURE 6:**
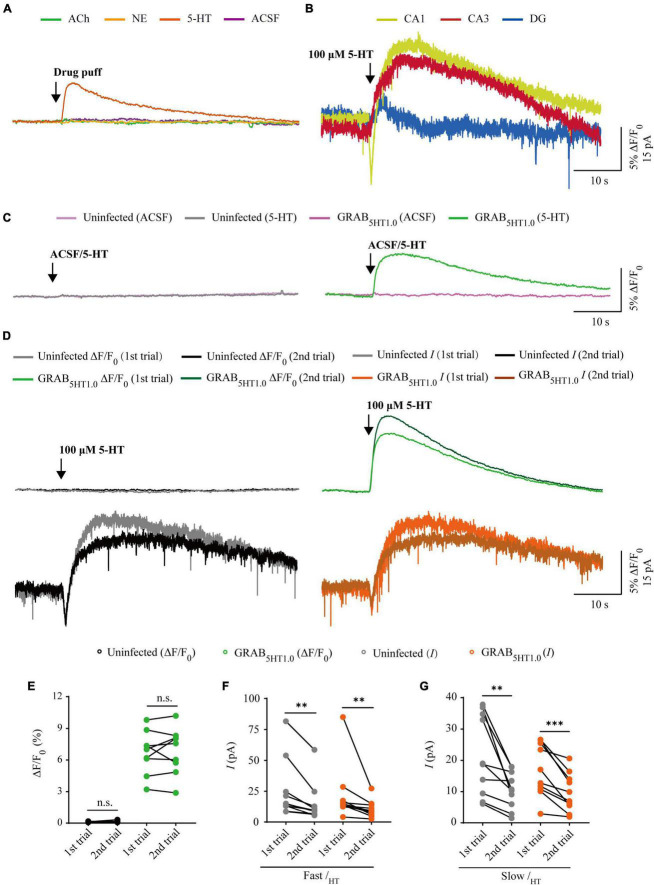
Comparison of GRAB_5HT1.0_ fluorescence and electrophysiological responses to two consecutive serotonin application. **(A)** Fluorescence responses of GRAB_5HT1.0_-expressing cells to a brief drug puff (10 ms) application of 100 μM 5-HT, 10 mM ACh, 200 μM NE, and ACSF, respectively. **(B)** Electrophysiological responses of CA1, CA3, and DG neurons to a brief (10 ms) puff application of 100 μM 5-HT. **(C)** Fluorescence responses of control non-expressing (left) and GRAB_5HT1.0_-expressing (right) CA1 neurons to a brief puff (10 ms) application of ACSF and 100 μM 5-HT. **(D)** Fluorescence (upper panel) and electrophysiological (lower panel) responses of non-expressing (left) and GRAB_5HT1.0_-expressing (right) CA1 neurons to two consecutive brief puff of 5-HT. **(E)** Values for the two consecutive fluorescence responses of control non-expressing CA1 neurons (first: 0.13 ± 0.01%; second: 0.14 ± 0.03%; *p* = 0.51; *n* = 9) and GRAB_5HT1.0_-expressing CA1 neurons (first: 6.70 ± 0.68%; second: 6.87 ± 0.73%; *p* = 0.65; *n* = 9). **(F)** Values for the two consecutive fast inward serotoninergic currents in control non-expressing (first: 25.47 ± 7.52 pA; second: 15.30 ± 5.12 pA; *p* = 0.0020; *n* = 10) and GRAB_5HT1.0_-expressing (first: 21.88 ± 7.23 pA; second: 10.63 ± 2.22 pA; *p* = 0.0020; *n* = 10) CA1 neurons. **(G)** Values for the two consecutive slow outward serotoninergic currents of control non-expressing (first: 21.63 ± 4.07 pA; second: 10.61 ± 1.88 pA; *p* = 0.0062; *n* = 10) and GRAB_5HT1.0_-expressing (first: 16.76 ± 2.64 pA; second: 9.73 ± 1.95 pA; *p* < 0.0010; *n* = 10) CA1 neurons. Data are shown as mean ± SEM. ^***^p < 0.001, ^**^p < 0.01, two-tailed Student’s paired *t*-test.

There are two types of genetic fluorescent sensors for 5-HT, including GRAB_5*HT*1_._0_ and iSeroSnFR. GRAB_5*HT*1_._0_ is constructed based on the serotonin 2C receptor; whereas iSeroSnFR is generated using PBP as serotonin recognition moiety (Unger et al., 2020; Wan et al., 2021). Previously we directly compared GRAB_5*HT*1_._0_ with iSeroSnFR in cultured rat hippocampal slices/mouse dorsal raphe neurons and found that GRAB_5*HT*1_._0_ showed significantly larger fluorescence changes (ΔF/F_0_) compared to iSeroSnFR (Zhang et al., 2022). Therefore, we chose GRAB_5*HT*1_._0_ as the genetically encoded serotonin sensor in our subsequent experiments. To compare GRAB_5*HT*1_._0_ sensor imaging with patch-clamp recordings for their detection ability for 5-HT, we simultaneously made dual whole-cell recordings and fluorescence imaging from pairs of neighboring GRAB_5*HT*1_._0_-expressing and non-expressing CA1 pyramidal neurons (Figure 5A), which had both inward and outward currents (Figure 6B), probably due to high expression of ionotropic and metabotropic receptors, such as 5-HT(3), 5-HT(1A), 5-HT(4), and 5-HT(7) subtypes (Bickmeyer et al., 2002; Eydipour et al., 2017). A 10 ms 5-HT (100 μM) puff evoked a brief, small inward current followed by a prolonged outward current in both GRAB_5*HT*1_._0_-expressing and non-expressing neurons, presumably representing the activation of endogenous ionic and G-protein coupled 5-HT receptors, respectively (Figures 5B, D). A concurrent fluorescence signal was observed only in GRAB_5*HT*1_._0_-expressing neurons but not in control non-expressing CA1 neurons (Figures 5B, C). The SNR of the GRAB_5*HT*1_._0_ fluorescence responses (∼65) seemed to be larger than that of the fast (∼13) and slow serotoninergic currents (∼12) (Figure 5E). The latencies of the fast serotoninergic currents were longer than those of the fluorescence responses (Figure 5F), indicating that GRAB_5*HT*1_._0_ detected 5-HT signals faster than endogenous serotoninergic receptors. To further compare the temporal responses, we compared rise time and decay time and found that GRAB_5*HT*1_._0_ showed both much slower rise but not decay time when compared to adrenergic currents of ionotropic and metabotropic receptors (Figures 5G, H), suggesting a little slower kinetics of GRAB_5*HT*1_._0_ over whole-cell patch clamp in reporting 5-HT signals. There was no significant difference in the amplitude, latency, SNR, rise time or decay time of serotoninergic currents in GRAB_5*HT*1_._0_-expressing and non-expressing neurons (Figures 5D–H), further confirming that the expression of fluorescence sensor GRAB_5*HT*1_._0_ did not change the intrinsic properties of the CA1 neurons. We further checked the specificity of GRAB_5*HT*1_._0_ and found that this sensor only showed fluorescent response to puffed 5-HT, but not ACh, NE, or control ACSF (Figure 6A). Moreover, we only detected fluorescent response in GRAB_5*HT*1_._0_-expressing neurons to puffed 5-HT, but not nearby control uninfected neurons to 5-HT/ACSF (Figure 6C), confirming the good specificity of GRAB_5*HT*1_._0_. Similar with what we observed with iAChSnFR and GRAB_*NE*_, the second 5-HT puff evoked the same fluorescence responses but smaller currents (reduced by ∼42%) in GRAB_5*HT*1_._0_-expressing neurons compared to the first puff (Figures 6D–G), due presumably to the desensitization of endogenous receptors (Berger et al., 2009), indicative of the suitability of GRAB_5*HT*1_._0_ in monitoring puffed 5-HT or presynaptic 5-HT release repeatedly over long periods.

## Discussion

In this study, we compared the performance of whole-cell patch clamp recordings and three genetically encoded fluorescence sensors, namely, iAChSnFR (Borden et al., 2020), GRAB_*NE*_ (Feng et al., 2019), and GRAB_5*HT*1_._0_ (Wan et al., 2021), in detecting ACh, NE, and 5-HT in cultured rat hippocampal slices. We conducted simultaneous electrophysiology recordings and fluorescence sensor imaging in hippocampal slices because they maintained the general synaptic organization and connections of the brain, which were ideal for repeated imaging and electrophysiology experiments (Jing et al., 2018; Borden et al., 2020). We tested the sensors in different regions of the hippocampus, including CA1, CA3, and DG, and found differences in cholinergic, adrenergic, and serotoninergic currents (Figures 2B, 4B, 6B), but not fluorescence differences among these regions. As a result, we chose CA3 neurons for ACh detection and CA1 neurons for NE/5-HT detection. We also tried to puff different concentrations of ACh/NE/5-HT and found that genetically encoded sensors, but not whole-cell recordings, responded to very low concentrations of neurotransmitters (Feng et al., 2019; Borden et al., 2020; Wan et al., 2021; Zhang et al., 2022). We optimized the concentrations of ACh, NE, and 5-HT to 10 mM, 200 μM, and 100 μM, respectively, at which concentrations both fluorescence and electrophysiology responses could be detected. Moreover, we also found all the neurotransmitter sensors had very good specificity. For instance, iAChSnFR only showed fluorescent responses to puffed ACh, but not NE, 5-HT, and ACSF (Figure 2A), and this also applied to GRAB_NE_ and GRAB_5HT1_._0_ (Figures 4A, 6A).

A 10 ms drug puff evoked robust fluorescence changes and inward/outward current in sensor-expressing neurons, representing the detection of neurotransmitters and the downstream activation of endogenous neurotransmitter receptors. Furthermore, we compared the responses of the fluorescence sensors and electrophysiological recording to two consecutive drug applications. The second ACh/NE/5-HT puff evoked the same fluorescence responses, but smaller currents in iAChSnFR/GRAB_NE_/GRAB_5HT1_._0_-expressing neurons compared to the first puff, probably due to the desensitization of endogenous receptors. However, the genetically encoded sensors did not trigger downstream receptor endocytosis signaling pathways, probably as a result of the mutations brought in the process of sensor constructions, making them ideal for monitoring puffed ACh/NE/5-HT or presynaptic neurotransmitter release over a long period repeatedly. Thus, these sensors permitted stable real-time measurements of the dynamic presynaptic release of the neurotransmitters. For example, iAChSnFR was used to visualize short-term depression of ACh vesicle release in mouse MEC neurons and 10 s was calculated as the time to relieve cholinergic presynaptic depression (Borden et al., 2020). Furthermore, combined with super-resolution and deconvolution microscopy, the genetically encoded sensors enabled the investigation of the fundamental properties of neurotransmission, such as the transmitter diffusion range and found that acetylcholine and monoamines employed spatially restricted transmission mode and diffused at individual release sites with a spread length constant of ∼0.75 μm (Zhu et al., 2020). When combined with high-resolution imaging and analysis algorithms, the genetically encoded sensors will unveil more properties of neurotransmitters such as the number of release sites, release pool size, release probability, quantal size, and refilling rate, that are crucial for comprehending the underlying mechanisms of behaviors and neurological diseases (Zhu et al., 2020; Chen et al., 2021; Lin et al., 2021; Zheng et al., 2022).

On the other hand, the genetically encoded neurotransmitter sensors have limitations and drawbacks that need to be considered when using them in future research studies. The kinetics of the newly developed genetically encoded neurotransmitter sensors is only rapid enough to report slow neurotransmitter releases generated by low- or moderate- but not high-frequency physiological stimulation. For example, both GACh2.0 and iAChSnFR failed to accurately report neurotransmitter release under high-frequency stimulation (>1 Hz) due to their long decay time (Jing et al., 2018; Borden et al., 2020). Moreover, the performance of the GPCR-based sensors can also be affected by agonists or antagonists that normally bind to their corresponding endogenous GPCRs, and this may limit the application of these sensors in certain pharmacological studies such as drug screening (Wu et al., 2022). In addition, the majority of PBP-based sensors, such as iSeroSnFR, iGABASnFR, and iATPSnFR (Lobas et al., 2019; Marvin et al., 2019; Unger et al., 2020), have relatively poor affinity for neurotransmitters because of their low sequence homogeneity to endogenous neurotransmitter receptors (Marvin et al., 2013). In addition, the ACh sensor iAChSnFR could detect both ACh and its precursor choline, limiting its application in high specificity requiring scenarios (Borden et al., 2020). Furthermore, the ectopic expression of genetically encoded sensors might interfere with endogenous signals since they are engineered neurotransmitter receptors and may differ from the endogenous receptor expression in some brain regions (Tubio et al., 2010; Wu et al., 2022). In this way, the overexpression of the genetically encoded sensors might not be appropriate for analyzing the spatiotemporal organization of neurotransmitter release (Liu et al., 2021). Finally, despite their cell specificity, remarkable sensitivity and excellent spatiotemporal resolution, the genetically encoded sensors could only detect the neurotransmitters released to the extracellular space but not the downstream signals that reflect the valid response from the target cells triggered by the binding of neurotransmitters, which may not reflect the real state of cells and cause overstatement of the importance of neurotransmitters in some behavior and diseases.

The classical patch clamp recording has remarkable sensitivity and temporal resolution and is a powerful tool to study the transmission properties of glutamatergic and gamma-aminobutyric acidergic transmitters (Jackman and Regehr, 2017; Chen et al., 2021). This is no doubt that patch-clamp recording is still the most direct and effective way of studying electrical signals in the brain (Reyes, 2019). However, this electrophysiology application is unsuitable for cells with minimal or no neuromodulator-induced electrophysiological responses. In this scenario, G-protein-activated potassium channels (GIRK2) were virally overexpressed in different brain regions of interest to provide a rapid electrophysiological readout of neuromodulator receptor activation (Marcott et al., 2014; Mamaligas and Ford, 2016). Despite its high informativity, the application of electrophysiology has strict requirement for experimental conditions, involves a complex experimental procedure, and is labor intensive but with low screening throughput (Sakmann, 2013; Wu et al., 2022). Moreover, both sensors and electrophysiological recordings only measure relative changes in ACh/NE/5-HT levels and do not provide concentration profiles. Fast-scan cyclic voltammetry (FSCV) is an electrochemical method commonly used to measure neuromodulator dynamics both *in vitro* and *in vivo* (Puthongkham and Venton, 2020; Venton and Cao, 2020). FSCV measures currents from direct oxidation of electroactive molecules and these currents are converted to concentrations using calibration values. Thus, genetically encoded sensors and whole-cell patch clamp recording could be combined with FSCV to study neurotransmitters dynamics in the future to understand their functional significance under both physiological and pathological conditions.

## Conclusion

In conclusion, the combination of different techniques for the study of neurotransmitters offers a valuable opportunity to gain new insights into the complex mechanisms of synaptic communication in the central nervous system. The classical patch clamp recording and genetically encoded sensors each have their own unique advantages and limitations, and it is important to carefully consider these when selecting the best tool for a particular study. By combining these techniques, researchers can obtain a more comprehensive and in-depth understanding of neurotransmitter dynamics in both physiological and pathological conditions, and further advance our knowledge of the underlying mechanisms of synaptic transmission.

## Data availability statement

The raw data supporting the conclusions of this article will be made available by the authors, without undue reservation.

## Ethics statement

The animal study was reviewed and approved by the Animal Care and Use Committees at Shanghai Jiao Tong University School of Medicine and South China University of Technology.

## Author contributions

PZ, YZ, and AC conceived and supervised the project. KZ performed the experiments related to sensor cloning and virus preparation. YH performed the simultaneous patch-clamp and sensor imaging experiments in slices. KZ, YH, PZ, YZ, and AC wrote the manuscript with input and help from other members from YZ and AC Labs. All authors contributed to data interpretation and data analysis.
